# Brentuximab vedotin: axonal microtubule's Apollyon

**DOI:** 10.1038/bcj.2015.72

**Published:** 2015-08-28

**Authors:** S Mariotto, S Ferrari, M Sorio, F Benedetti, G Tridente, T Cavallaro, A Gajofatto, S Monaco

**Affiliations:** 1 Department of Neurological and Movement Sciences, University of Verona, Verona, Italy; 2 Department of Clinical and Experimental Medicine, University of Verona, Verona, Italy; 3 School of Medicine, University of Verona, Verona, Italy

Chemotherapy-induced peripheral neuropathy (CIPN) is one of the most common side effects encountered in patients treated with chemotherapeutic drugs binding to soluble tubulin or targeting microtubules.^1, 2^ Some of these drugs, such as taxanes and ixabepilone, stabilize and block microtubule remodeling, whereas others, including Vinca alkaloids, colchicine and eribulin, promote microtubule disassembly.^3^ Over the last few years, the use of antibody-drug conjugates (ADCs) with potent and highly specific activity against protein targets, which are abundantly expressed on malignant cells, has greatly improved the outcome of patients with cancer. Among ADCs used in patients with hematologic cancer, an anti-CD30 chimeric antibody conjugated by a protease-cleavable dipeptide to monomethyl auristatin E (MMAE; a synthetic agent that blocks polymerization of tubulin), named as brentuximab vedotin (BV), has demonstrated efficacy in the treatment of relapsed or refractory Hodgkin's lymphoma (HL) and systemic anaplastic large-cell lymphoma.^4, 5, 6^ Neurotoxicity is the greatest concern regarding BV, and the most frequent complication is peripheral neuropathy (PN), which often causes discontinuation of therapy.^6, 7, 8, 9, 10, 11^ The mechanisms responsible for neuropathy remain unknown, as peripheral nerves do not express CD30, thus apparently precluding the targeted delivery of MMAE to nerve fibers. However, it is unknown whether untargeted or systemic delivery of ADC and/or free MMAE to neuronal bodies and their axonal extensions may occur.^12^ To date, no studies are available on morphological and ultrastructural changes occurring in peripheral nerves of patients with BV-related PN. Among patients with HL in treatment with BV, who are referred to our clinic for neurological evaluation and electrophysiological investigations, we recently had the opportunity to study a patient with severe neuropathy and to investigate pathological changes at sural nerve biopsy.

A 22-year-old man affected with HL, diagnosed in 2009, was treated with doxorubicin, bleomycin, vinblastine and dacarbazine followed by BEACOPP, local mediastinal radiotherapy and autologous stem-cell transplantation. In January 2012, allogeneic bone marrow transplantation was performed, and on February 2013 he was started on BV at the standard intravenous dose of 1.8 mg/kg every 3 weeks for up to 16 cycles. After the seventh cycle, the patient developed progressive weakness of both legs and he walked only with forearm crutches. Two months later he began to complain of distal numbness in the hands and feet; nevertheless, the treatment was completed without dose modification or delay. In March 2014, owing to rapid progression of weakness over the 2 months following completion of BV cycles, the patient was sent to our clinic for neurological evaluation and neurophysiological testing. Ambulation with crutches revealed a steppage gait, more severe on the right. On testing of muscle strength there was 3/5 strength with elbow and wrist flexion/extension on Medical Research Council scale, and 2/5 with hand grip; knee flexion and extension were 3/5, ankle dorsiflexion showed 0/5 strength and ankle flexion 1/5. The patient had paresthesias and dysethesias in both hands and feet; sensory responses to light touch, pinprick and temperature were intact. There was decreased vibratory sensation in a bilateral glove-and-stocking distribution, more marked distally. The deep-tendon reflexes were abolished in the lower and upper limbs. Neurophysiological testing showed changes consistent with severe predominantly motor neuropathy; the peroneal and tibial motor responses were not recordable, whereas reduced sensory sural nerve responses were obtained on both sides. Motor nerve conduction studies yielded a mild increase in distal motor latencies of median nerves with severe reduction of compound muscle action potential, more on the right, whereas ulnar nerves were less affected. The sensory conduction studies of the median and ulnar nerves displayed reduced amplitudes and nearly 30% reduction of normal age values in conduction velocities (CVs).

Given the rapid progression and severity of the neuropathy and the findings of nerve conduction studies, a sural nerve biopsy was performed. Plastic-embedded sections of the biopsy specimen showed features of active axonal neuropathy with decreased density of myelinated fibers of all calibers, ongoing wallerian-like degeneration and sporadic small clusters of regenerating fibers, in the absence of cellular inflammation (Figure 1a). Electron microscopical examination revealed alterations in axonal cytoskeleton, altered orientation of microtubules (MT) and severe decrease in identifiable MT profiles in myelinated (Figures 1b and c), and to a lesser extent, in unmyelinated fibers, as compared with control axons (Figure 1d). Determination of the number of MT was obtained by direct count in a total of 30 myelinated axons from the patient's sural nerve and 30 control axons, as previously described.^13^ After counting all MT in randomly sampled axons, we found that the median MT density was 1.3/μm^2^ (range 0.3–7.6) in the case index, as opposed to 9.9/μm^2^ (range 7.1–17.5) in control fibers. In addition to MT loss, some axons showed a spatial disorganization of neurofilaments and a variable increase in smooth endoplasmic reticulum and membranous organelles, findings suggestive of an impairment of fast anterograde transport. Over the next 5 months, an improvement in ambulation and muscle strength in both hands was observed, with restoration of biceps and triceps reflexes; decrease in paresthesias was also reported. An improvement in motor and sensory functions, mostly in the upper limbs, was further observed 7 months later, in keeping with neurophysiological findings revealing an increase of nerve CVs and amplitudes.

During the course of treatment with BV, the patient here reported developed sensorimotor axonal neuropathy, a complication that was somewhat expected given its reported high frequency in patients exposed to BV and polatuzumab vedotin.^6, 9, 14^ The onset was subacute and characterized by slightly asymmetric weakness that predominantly affected lower extremity extensors, leading to foot drop and impaired ambulation, in addition to involvement of upper extremities with marked reduction of grip strength. Within few weeks, motor events were followed by sensory manifestations, including paresthesias and loss of large fiber sensory modalities in a length-dependent pattern. No apparent autonomic dysfunction or light touch and pinprick loss were observed, a finding suggestive of sparing of small fibers. Fourteen months after all 16 cycles were completed, a slight improvement of signs and symptoms was noted, although the occurrence of muscular atrophy and sensory loss are likely to be considered permanent events. The absence of conditions predisposing to PN, in addition to the occurrence of manifestations during treatment and the temporal evolution of the sensorimotor changes, suggest a causal association between BV administration and the neurotoxic complication; nevertheless, the contribution of antecedent chemotherapies cannot be completely ruled out. Similarly to other CIPN, BV-induced PN is characterized by a length-dependent axonopathy, with early changes occurring at the distal sites and ensuing spread to more proximal locations, a pattern coherent with the dying back model. At variance with previous investigations, reporting a high frequency of sensory symptoms at neuropathy onset,^7, 8, 9^ in our case the presentation was that of a motor axonal neuropathy with asymmetric features. Indeed, sensory signs and symptoms occurred only several weeks later, thus suggesting that still unknown factors may have a role in the selective vulnerability of different neuronal populations. The occurrence of graded, symmetric sensory loss with a length-dependent pattern, in addition to the detection of small clusters of regenerating fibers in the obtained nerve biopsy, is further indicative of distal axonal degeneration, and apparently rules out a neurotoxic effect at the level of sensory neurons residing in dorsal root ganglia. Although the exact mechanisms of BV-induced PN remain to be elucidated, it is reasonable to suggest that damage to peripheral axons may be the consequence of its effects on MT polymerization. This hypothesis is supported by pathological changes encountered in PN induced by vincristine, a Vinka alkaloid drug that impedes polymerization of tubulin into MT. Indeed, previous experimental work has demonstrated that in vincristine-induced PN there is a decreased total number and density of axonal MT in myelinated sensory axons, in addition to altered MT orientation and arrangement.^15^ These MT abnormalities interfere with fast axonal transport, an intracellular bidirectional system devoted to the anterograde supply of cytoskeletal/membrane components, organelles and protein particles to axon terminals, and retrograde delivery of endosome and gene expression signals to the neuronal cell body. A comparable effect of BV and other ADCs bearing vedotin has been claimed in the pathogenesis of PN, however, no investigations are available to date. Although the pathological study of a single case represents a major limitation of our report, the present demonstration of severe MT loss in myelinated fibers of the sural nerve in a patient with BV-associated PN, strongly confirms that the neurotoxicity of this ADC is exerted through disruption of axonal MT. In addition to MT loss, some axons showed a spatial disorganization of neurofilaments and variable increase in smooth endoplasmic reticulum and membranous organelles, findings suggestive of an impairment of fast anterograde transport. How peripheral nerves are exposed to antibody-conjugated MMAE or free MMAE remains to be clarified. In previous studies, a similar pharmacokinetic profile for vedotin-bearing ADCs and unconjugated MMAE was found, with steady state occurring by 21 days; notably, the concentration of BV was much higher than MMAE.^8^ Therefore, untargeted delivery or diffusion of BV to peripheral nerves, or a bystander effect, following detachment of MMAE from the antibody, may account for exposure of the axonal cytoskeleton to this microtubule's ‘Apollyon'.

## Figures and Tables

**Figure 1:**
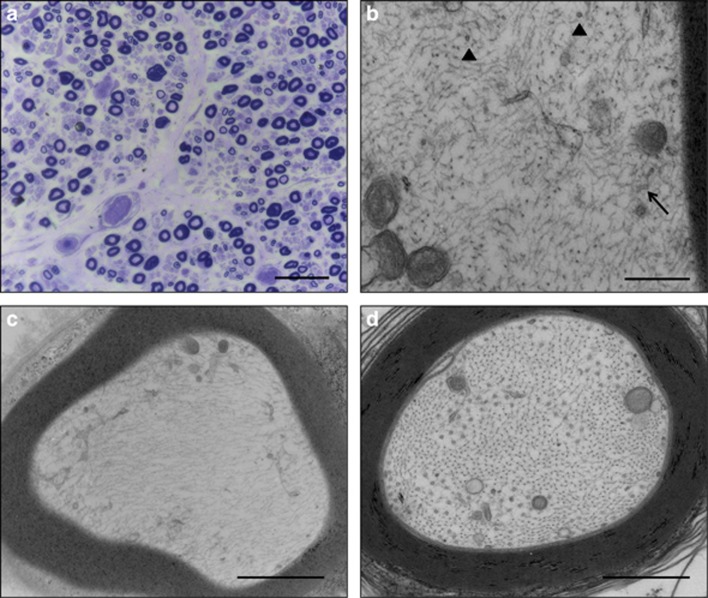
(**a**) A Spurr-embedded section of the sural nerve biopsy specimen shows reduction in density of myelinated fibers, degenerating axons and a few regenerating clusters, findings consistent with ongoing axonal neuropathy (scale bar=50 μm). (**b**) High magnification electron micrograph of a myelinated axon of the patient's sural nerve shows a few microtubule profiles oriented along the longitudinal axis of the fiber (arrowhead), in addition to tangentially oriented microtubules (arrow; scale bar=300 nm). (**c**) A myelinated fiber showing severe depletion of microtubules, bundles of misaligned neurofilaments and subaxolemmal segregation of smooth endoplasmic reticulum and membranous organelles (scale bar=120 nm). (**d**) A control myelinated fiber showing the typical alignment of the axonal cytoskeleton and the distribution of microtubules in distinct microdomains (scale bar=120 nm).
